# The Dark Side of Microbial Processes: Accumulation of Nitrate During Storage of Surface Water in the Dark and the Underlying Mechanism

**DOI:** 10.1128/spectrum.02232-21

**Published:** 2022-01-05

**Authors:** Amit Kumar, Daphne H. P. Ng, Sakcham Bairoliya, Bin Cao

**Affiliations:** a School of Civil and Environmental Engineering, Nanyang Technological University, Singapore; b Singapore Centre for Environmental Life Sciences Engineering, Nanyang Technological University, Singapore; University of Minnesota

**Keywords:** microbial processes, nitrifying bacteria, water quality

## Abstract

In densely populated cities with limited land, storage of surface water in underground spaces is a potential solution to meet the rising demand of clean water. In addition, due to the imperative need of renewable solar energy and limited land resources, the deployment of floating solar photovoltaic (PV) systems over water has risen exponentially. In both scenarios, microbial communities in the water do not have access to sunlight. How the absence of sunlight influences microbial community function and the water quality is largely unknown. The objective of this study was to reveal microbial processes in surface water stored in the dark and water quality dynamics. Water from a freshwater reservoir was stored in the dark or light (control) for 6 months. Water quality was monitored at regular intervals. RNA sequencing was performed on the Illumina MiSeq platform and qPCR was used to substantiate the findings arising from the sequencing data. Our results showed that storage of surface water in the dark resulted in the accumulation of nitrate in the water. Storage in the dark promoted the decay of algal cells, increasing the amount of free nitrogen in the water. Most of the free nitrogen was eventually transformed into nitrate through microbial processes. RNA sequencing-based microbial community analyses and pure culture experiments using nitrifying bacteria Nitrosomonas europaea and Nitrobacter sp. revealed that the accumulation of nitrate in the dark was likely due to an increase in nitrification rate and a decrease in the assimilation rate of nitrate back into the biomass.

**IMPORTANCE** Microbial communities play an essential role in maintaining a healthy aquatic ecosystem. For example, in surface water reservoirs, microorganisms produce oxygen, break down toxic contaminants and remove excess nitrogen. In densely populated cities with limited land, storing surface water in underground spaces and deploying floating solar photovoltaic (PV) systems over water are potential solutions to address water and energy sustainability challenges. In both scenarios, surface water is kept in the dark. In this work, we revealed how the absence of sunlight influences microbial community function and water quality. We showed that storage of surface water in the dark affected bacterial activities responsible for nitrogen transformation, resulting in the accumulation of nitrate in the water. Our findings highlight the importance of monitoring nitrate closely if raw surface water is to be stored in the dark and the potential need of downstream treatment to remove nitrate.

## INTRODUCTION

According to the National Academy of Engineering, access to clean water is one of the 14 grand challenges for engineering in the 21st century (1). Rainwater, which generally accumulates as surface water in reservoirs, is an important source of clean water (2). Rapid increase in the world population is expected to give rise to a massive demand to harvest additional rainwater (3, 4). However, harvesting of rainwater can be challenging for densely populated cities with limited land space to spare for creating new surface reservoirs.

A potential solution to mitigating this problem is to build and use underground or subsurface storage systems for the storage of surface water. One major difference between surface reservoirs and subsurface storage systems is the absence of sunlight in the latter. Sunlight, especially light in the visible spectrum, is an important environmental factor shaping microbial communities in aquatic environments (5). Sunlight participates in various environmental processes and drives biogeochemical cycles, influencing the physicochemical quality of the resident water (6). For example, the visible spectrum of sunlight stimulates the growth of phototrophic microorganisms, while some filamentous fungi thrive in the dark (7). As visible light is the source of energy for photosynthesis, all organisms in the ecosystem ultimately depend on primary producers to convert solar energy into chemical energy. How the microbial community function and the quality of the water stored in the dark conditions differ from those of water in surface reservoirs remains unknown.

In addition, due to the imperative need of renewable solar energy and limited land resources, the deployment of floating solar photovoltaic (PV) systems over water has risen exponentially (8). The impact of floating PV systems on aquatic ecosystems has been mainly focused on temperature and dissolved oxygen as well as effects on aquatic flora and fauna (9–11). How the dark environment created by the floating structures affects the microbial community function and the quality of the water remains unknown.

The objective of this study was to reveal microbial processes in surface water stored in the dark and water quality dynamics. Water from a freshwater reservoir was stored in the dark for 6 months with water from the same source stored in the light serving as a control. Water quality was monitored, and microbial community function was examined using RNA sequencing and qPCR analyses. Transformation of nitrogen in the water stored in the dark and light conditions was compared. In addition, nitrifying bacterial cultures of Nitrosomonas europaea and Nitrobacter sp. were used to validate the proposed mechanism.

## RESULTS AND DISCUSSION

### Water quality dynamics.

**FIG 1:**
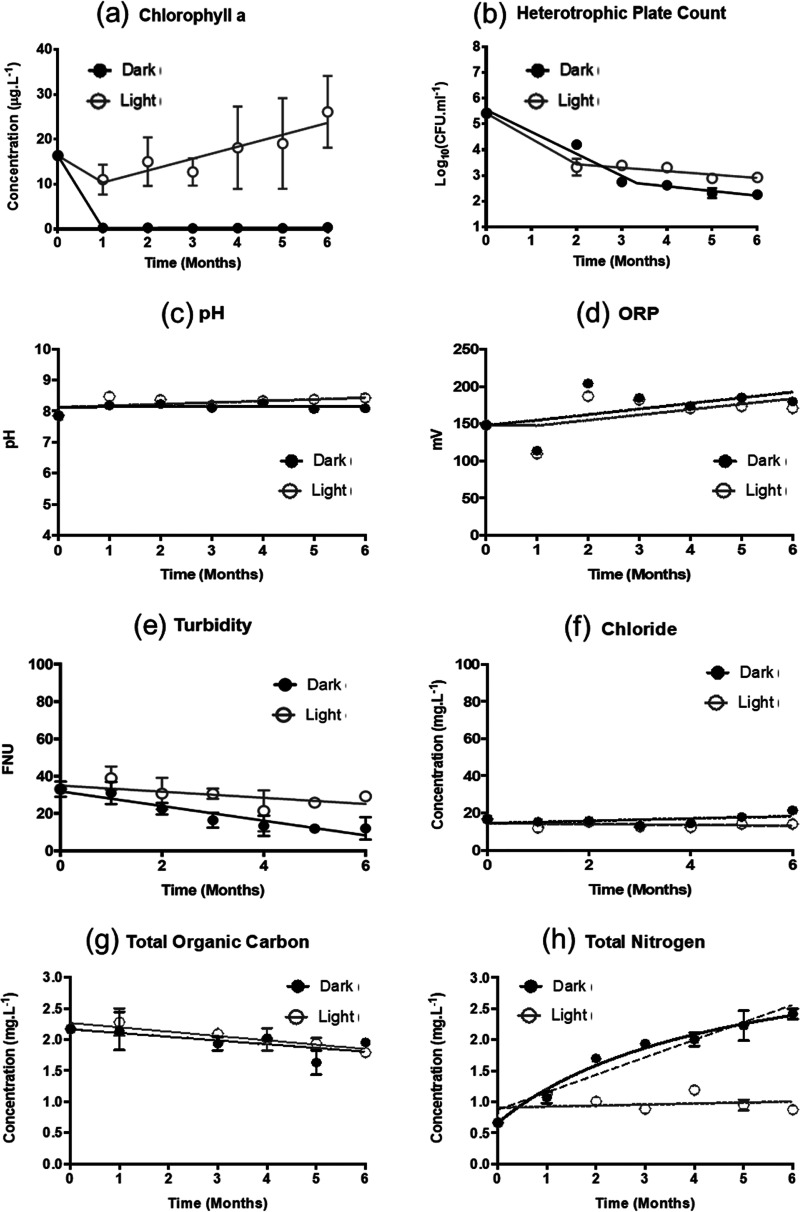
Changes in water quality parameters of the surface water stored in the dark and light. (a) Total algae (measured as chlorophyll *a*) decreased rapidly in the dark within a month. (b) The decay of heterotrophic bacteria was biphasic in both light and dark. (c, d, e, f, g) The pH, ORP, chloride, and total organic carbon (TOC_aq_) remained relatively uniform both in light and dark, while there was a considerable decrease in the turbidity. (h) Total nitrogen (TN_aq_) increased in the dark over time and plateaued eventually. Dotted line represents the linear fitting of TN_aq_ values in the dark.

For water stored in the dark, total algae (measured as chlorophyll *a* content) decreased rapidly from 16.4 ± 1.5 μg/L to 0.4 ± 0.3 μg/L within a month (Fig. 1a). Light as a source of energy is essential to support and maintain the population of photosynthetic algae and cyanobacteria in the ecosystem (12). This observation was also corroborated by the observation that in the water stored in the light, the concentration of total algae maintained at a high level throughout the experiment and increased from 16.4 ± 1.5 μg/L to 26.1 ± 13.8 μg/L in 6 months.

The heterotrophic plate count (HPC) of bacteria in the water was determined to be 2.5 ± 0.5 × 10^5^ CFU/mL and decreased in both the light and the dark conditions (Fig. 1b). After 6 months, the HPC of the water in the light (7.9 ± 0.2 × 10^2^ CFU/mL) was significantly higher (*P* < 0.05) than that in the dark (2.0 ± 0.2 × 10^2^ CFU/mL). The decay of HPC in the water occurred in two distinct phases with a faster initial decay followed by a subsequent slow rate of decay. In the light, the initial rate of decay was approximately 1.6 × 10^5^ CFU/mL/month while the rate of decay in the second phase was about 10 times lower. Similarly, the initial rate of decay in the dark was approximately 1.1 × 10^5^ CFU/mL/month while the rate of decay in the second phase was about 5 times lower. Previous studies have also shown biphasic decay of microorganisms (13, 14), which is attributed largely to the population heterogeneity and the ability of some bacterial cells to persist in starvation conditions at low cell densities (14, 15). Besides, the presence of algae and cyanobacteria may have contributed to the higher HPC in the light compared to that in the dark as heterotrophic microorganisms may depend on the metabolites produced by the primary producers for growth and maintenance (16).

The total organic carbon (TOC; ∼2.2 mg/L), pH (∼8.0), oxidation-reduction potential (ORP; ∼150 mV), and chloride content (∼17 mg/L) were comparable for water stored in the dark and light throughout the experiments (Table 1 and Fig. 1). In contrast, the turbidity of the water reduced substantially both in the light (5.2 ± 1.8 FNU/month) and dark (6.6 ± 1.1 FNU/month). Decay of total algae in the dark (Fig. 1a) could have contributed to the lower turbidity of the water in the dark compared to the light (Fig. 1e). The total nitrogen (TN_aq_) content of the water stored in the light remained close to its initial value (∼0.7 mg/L) over the course of the experiment. However, in the dark, the TN_aq_ content increased significantly (*p* < 0.05) from 0.7 ± 0.0 mg/L to 2.4 ± 0.1 mg/L in six months (Fig. 1h). The rate of change of TN_aq_ in the dark was 0.3 ± 0.0 mg/L/month, while no change of TN_aq_ was observed in the presence of light.

**TABLE 1:** Average rate of change for chloride (mg.L^−1^/month), pH (/month), ORP (mV/month), turbidity (FNU/month), TOC (mg.L^−1^/month), and TN (mg.L^−1^/month) in dark and light samples^*a*^

	Dark		Light		Light vs dark
Water quality parameters	Avg rate of change	*P* value^*b*^ (w.r.t. slope zero)	Avg rate of change	*P* value (w.r.t. slope zero)	*P* value (light vs dark)
Chloride	0.64 ± 0.28	0.038 (NS)	–0.23 ± 0.28	0.426 (NS)	0.02 (NS)
pH	0.02 ± 0.02	0.300 (NS)	0.05 ± 0.02	0.017 (NS)	0.14 (NS)
ORP	7.45 ± 2.69	0.012 (NS)	6.38 ± 2.44	0.017 (NS)	0.64 (NS)
Turbidity	–6.60 ± 1.15	<0.001 (S)	–5.19 ± 1.84	0.014 (NS)	0.33 (NS)
TOC_aq_	–0.06 ± 0.03	0.065 (NS)	–0.07 ± 0.02	0.058 (NS)	0.66 (NS)
TN_aq_	0.33 ± 0.02	<0.001 (S)	0.07 ± 0.01	0.434 (NS)	0.0003 (S)

a NS, nonsignificant; S, significant.

b For all cases except turbidity and TN_aq_ in the dark, the *P* value with respect to (w.r.t.) slope zero is nonsignificant, i.e., the slope is not significantly nonzero (*P* < 0.008). The change of slope between light and dark was significantly different (*P* < 0.008) only for TN_aq_.

### Nitrate accumulation in the dark condition.

**FIG 2:**
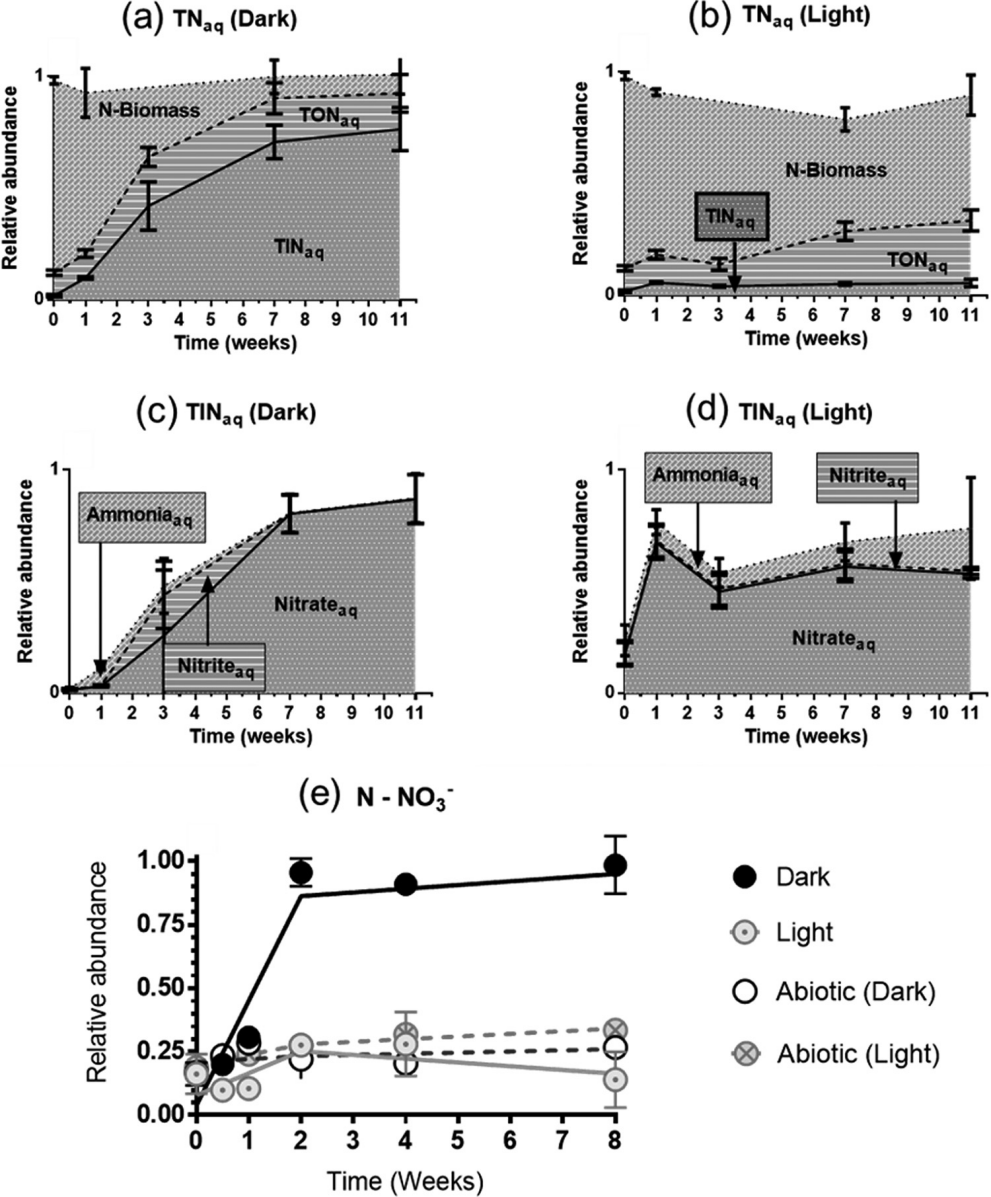
Distribution of N in the water stored in the dark and light. At the start of the experiment, most of the N was in the form of biomass (N-biomass). (a) In the dark, N-biomass decreased and total inorganic nitrogen (TIN_aq_) increased over time. (b) In the light, the decrease in N-biomass was not as prominent as in the dark. (c) Almost all TIN_aq_ was in the form of nitrate in the dark. (d) Most of the TIN_aq_ was in the form of nitrate in the light although the percentage of TIN_aq_ was very low. (e) Accumulation of nitrate in the dark was a microbial process as the concentration of nitrate did not increase in the abiotic samples stored in the dark or light.

To further elucidate the processes of N accumulation in the water under the dark condition, mass balance was conducted. At the start of the experiment, most of the N (∼88%) was in the form of biomass, that is, N-biomass, while soluble organic N (TON_aq_) and inorganic N (TIN_aq_) contributed to ∼11% and 1% of total N, respectively (Fig. 2). In the presence of light, a marginal decrease in the N-biomass was observed, and the TON_aq_ increased from 11% to 28% in 11 weeks. An increase in TIN_aq_ from 1% to 5% was also observed immediately after the start of the experiment and then remained relatively constant (Fig. 2b). In the dark condition, N-biomass decreased rapidly over a period of 11 weeks from 88% to 8%. Although a marginal increase in TON_aq_ was observed (from 11% to 15%), the decrease in N-biomass mainly led to the increase in TIN_aq_ from 1% to 76% (Fig. 2a). These results revealed that the increase in the concentration of aqueous nitrogen (TN_aq_) in the dark was coupled with the decrease in the nitrogen present in the biomass. In addition, there was a marginal change in TON_aq_ over time and the decrease in the N-biomass correlated well with the increase in TIN_aq_.

Further experiments revealed that nearly all of the soluble inorganic nitrogen was in the form of nitrate (Fig. 2c). Nitrate was also the major constituent (>80%) of the TIN_aq_ in the light (Fig. 2d) despite the observation that TIN_aq_ under this condition was very low (1%) (Fig. 2b). A small fraction of ammonia (<20%) was present in the light while nitrite was below the detection limit (0.02 mg/L NO_2_-N) throughout the experiment. In the dark, nitrate (>99%) was the dominant constituent of TIN_aq_ in the later stages of the experiment. Although ammonia was the major fraction of the inorganic nitrogen at the start of the experiment and a small amount of nitrite accumulated around weeks 2–7, these two species were not detected in the later stages (after 7 weeks) of the experiment. Hence, under the dark condition, the decrease of N in the biomass correlated to the increase in the concentration of nitrate, while other inorganic forms of aqueous nitrogen such as nitrite and ammonia were present in negligible or marginal concentrations throughout the experiment. As expected, no substantial changes were observed in the nitrate concentrations of the abiotic control stored under either the light or the dark conditions (Fig. 2e), suggesting that the conversion of N in the biomass to inorganic nitrogen, in particular nitrate, was a biotic process.

### Role of microorganisms in nitrate accumulation in the dark.

To examine the role of microorganisms in the accumulation of nitrate in the dark, we sequenced total RNA extracted from the water samples. RNA sequencing was conducted to investigate the community composition at a time point when the physicochemical differences among the light and dark samples were greatest (i.e., at the end of the experiment). As microorganisms are the key drivers of biogeochemical cycles in ecosystems, the differences in the microbial communities in water stored in the light and dark provide an explanation for the physicochemical changes observed in the water. The sequencing statistics can be found in Table S1 in the supplemental material.

**FIG 3:**
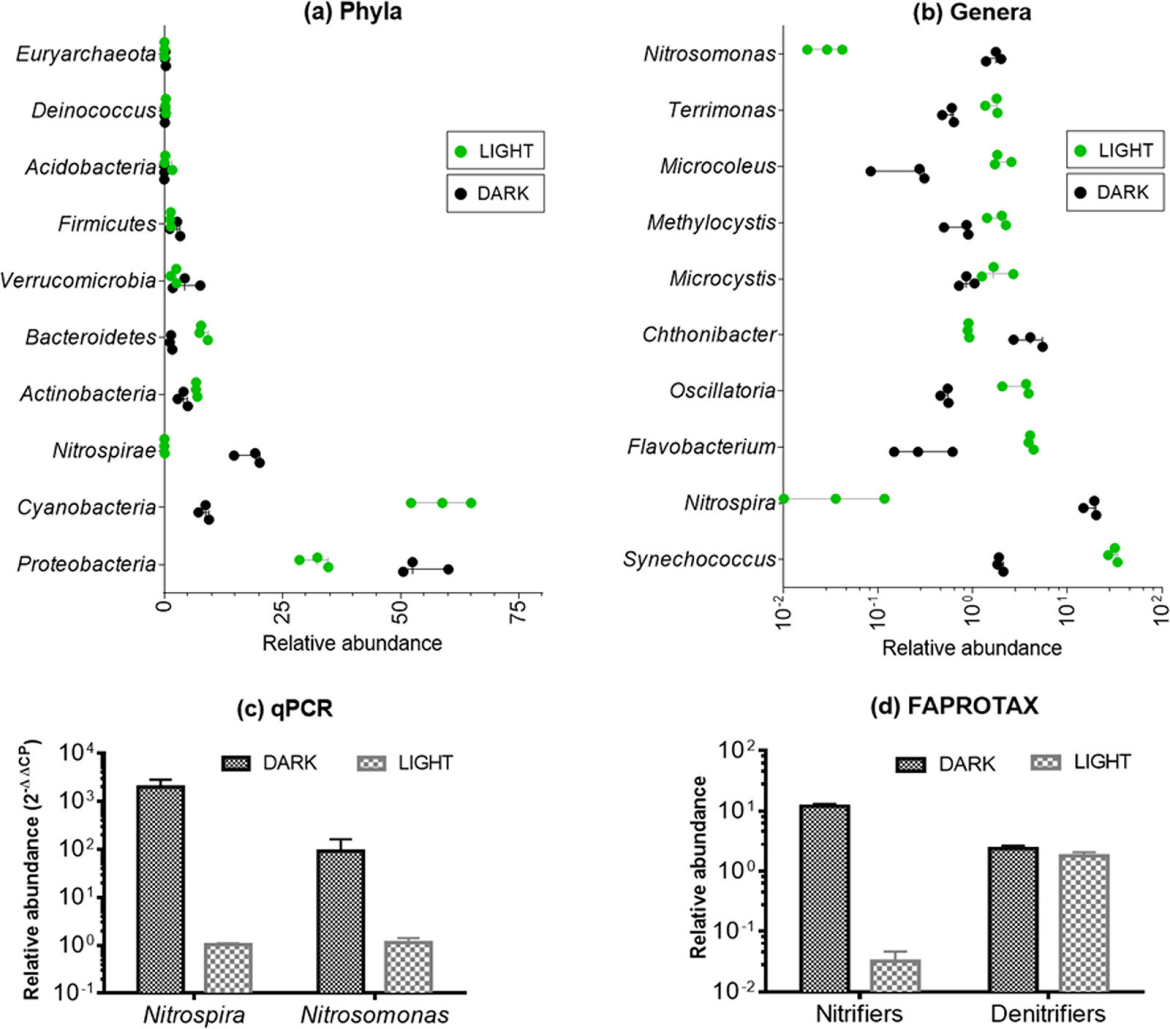
Analysis of 16S rRNA sequencing of the water samples from light and dark. (a) *Proteobacteria* was well represented in both light and dark. *Cyanobacteria* was the most abundant phyla in the light and scarce in dark. *Nitrospirae* was much more abundant in the dark compared to light. (b) *Synechococcus* was the most abundant genera in the light. In the dark, *Nitrospira* was the most abundant genera and almost absent in the light. (c) The qPCR results also confirmed that the nitrifying bacteria *Nitrospira* and *Nitrosomonas* were much more abundant in the dark than in the light. (d) Functional annotation based on taxonomy revealed that the relative abundance of all the nitrifiers was about 400-fold higher in the dark compared to the light, while the abundance of denitrifiers was nearly the same.

Analysis of the 16S rRNA sequences of the water stored under the light and the dark conditions revealed the phylum *Proteobacteria* as the most abundant bacterial group in the dark and the second most abundant group in the light (Fig. 3a). *Cyanobacteria* was the most abundant phylum in the water stored in the light, and it was scarce in the water from the dark condition. In addition, *Actinobacteria* and *Bacteroidetes* were also more abundant in the light condition. For the water stored in the dark, the relative abundance of the phylum *Nitrospirae* was 360-fold higher than that in the light.

Analysis of the sequencing data at the genus level revealed that *Synechococcus* in the phylum of *Cyanobacteria* was most abundant in the light, while *Nitrospira* was the most abundant genera in the dark (Fig. 3b). The relative abundance of *Nitrospira* and *Nitrosomonas* was approximately 1056-fold and 175-fold, respectively, in the dark compared to the light. The abundance of *Nitrosomonas* and *Nitrospira* observed in the sequencing data were verified by qPCR (Fig. 3c). The qPCR results showed that the gene copy numbers of *Nitrospira* were higher by more than 3 orders of magnitude in the dark samples compared to the samples from the light. The gene copy numbers of *Nitrosomonas* were also significantly higher (*P* < 0.01) for the dark samples compared to the light samples.

Previous studies have reported that bacteria from the phylum *Cyanobacteria* could be involved in the assimilation of inorganic nitrogen (17), while some bacterial genera from the phylum *Nitrospirae* are known to be involved in nitrification (18). For example, *Synechococcus* and other *Cyanobacteria* are known to drive assimilatory nitrate reduction in aquatic ecosystems (19, 20). *Nitrosomonas* and *Nitrospira* belong to the group of nitrifying bacteria and are responsible for converting ammonia to nitrite and nitrite to nitrate, respectively (21). *Nitrospira* has also been reported to be able to perform complete nitrification of ammonia to nitrate (22).

The reduction in the abundance of *Cyanobacteria* under dark conditions could have contributed to the reduced nitrate assimilation, while the increased relative abundance of nitrifying organisms (*Nitrosomonas* and *Nitrospira*) could have enhanced nitrification processes. Functional annotation of 16S rRNA sequences using FAPROTAX also revealed that the relative abundance of bacteria involved in nitrification was about 400-fold higher in the dark compared to the light (Fig. 3d), implying that nitrification was enhanced in the dark. In contrast, the relative abundance of bacteria involved in denitrification was comparable in both the light and dark conditions. Hence, the observed accumulation of nitrate in the water stored in the dark condition was likely attributed to enhanced nitrification activity in the dark.

### Effect of light on model ammonia-oxidizing and nitrite-oxidizing bacteria.

**FIG 4:**
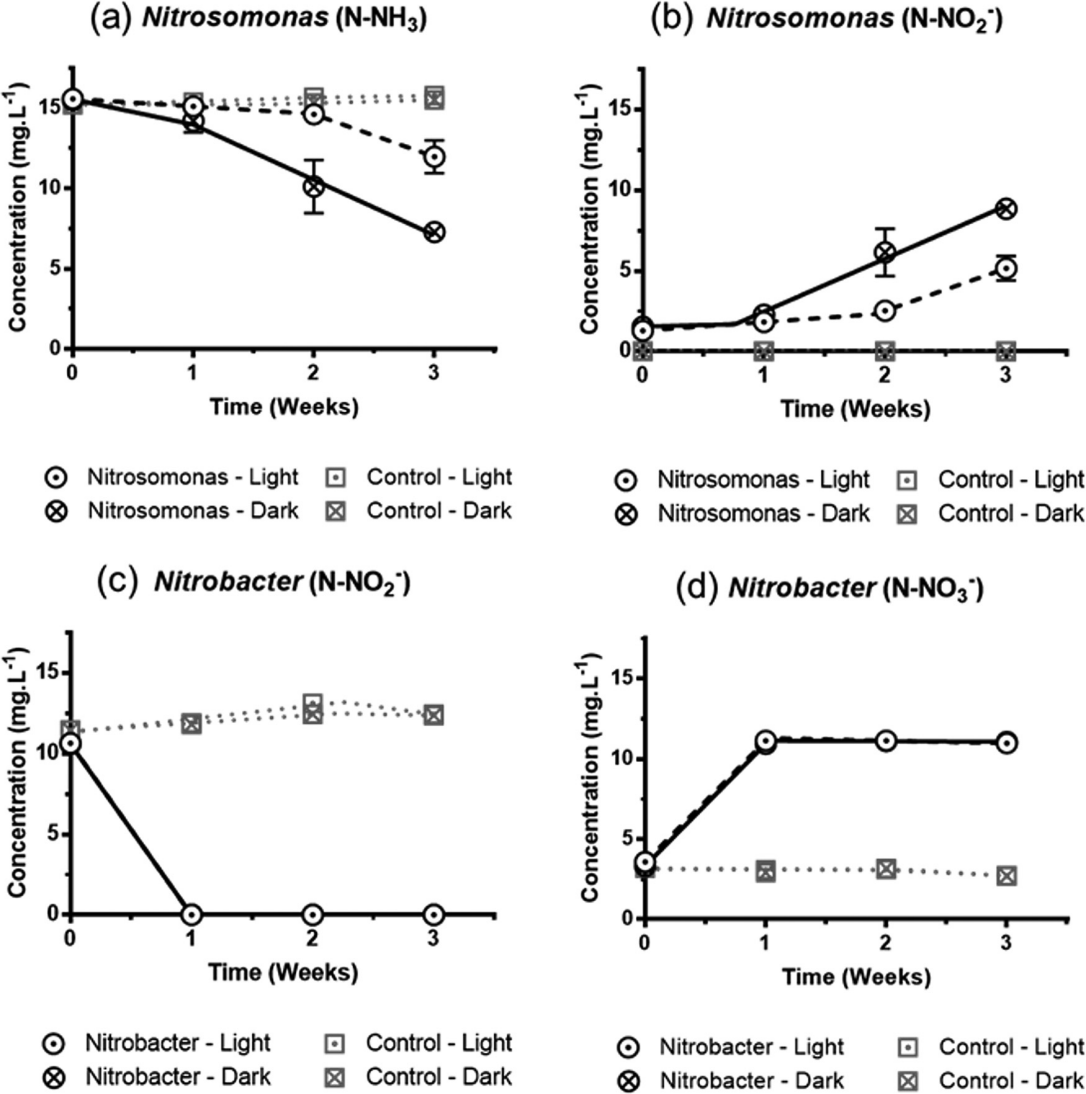
Nitrification experiments in the light and the dark using model organisms *Nitrosomonas* and *Nitrobacter*. (a) The consumption of ammonia and (b) the production of nitrite by *Nitrosomonas* were faster in the dark compared to light. (c) The consumption of nitrite (d) and the production of nitrate by *Nitrobacter* were not affected by the light and were similar to that in the dark. In both the light and the dark, the rate of nitrite to nitrate conversion by *Nitrobacter* was much faster than the rate of ammonia to nitrite conversion by *Nitrosomonas*.

To further test whether the nitrification activity could be enhanced in the dark condition, we examined the nitrification activity of pure cultures of a *Nitrosomonas* strain and a *Nitrobacter* strain. The consumption of ammonia by *Nitrosomonas* in the dark was significantly higher (*P* < 0.05) compared to that in the light (Fig. 4a). After 3 weeks, ammonia consumption by *Nitrosomonas* was approximately 2.5 times higher in the dark compared to the light. Meanwhile, the concentration of nitrite observed in the *Nitrosomonas* culture under the dark condition was 1.7 times higher (Fig. 4b). In contrast, there was no significant impact of light on the conversion of nitrite to nitrate by *Nitrobacter* (Fig. 4c and d). The conversion of nitrite to nitrate by *Nitrobacter* was significantly faster (*P* < 0.05) compared to the conversion of ammonia to nitrite by *Nitrosomonas*. Unlike the conversion of ammonia to nitrite by *Nitrosomonas*, nearly all the nitrite was converted to nitrate within a week by the *Nitrobacter* cultures in both the light and the dark. This result suggested that the nitrification in the light was limited by the conversion of ammonia to nitrite by *Nitrosomonas* instead of conversion of nitrite to nitrate by *Nitrobacter*.

Our results are consistent with previous reports on the inhibition of *Nitrosomonas* activity by light (23, 24). In addition, previous studies have reported that *Nitrobacter* activity is inhibited by light as well (25, 26). On the contrary, our observation suggested that light had no effect on the activity of nitrite to nitrate conversion by *Nitrobacter*. This could likely be due to the strain differences among the studies, as observed in a previous study which showed that some strains of *Nitrobacter* are less sensitive to light than others (27).

### Proposed mechanism for nitrate accumulation in the dark.

**FIG 5:**
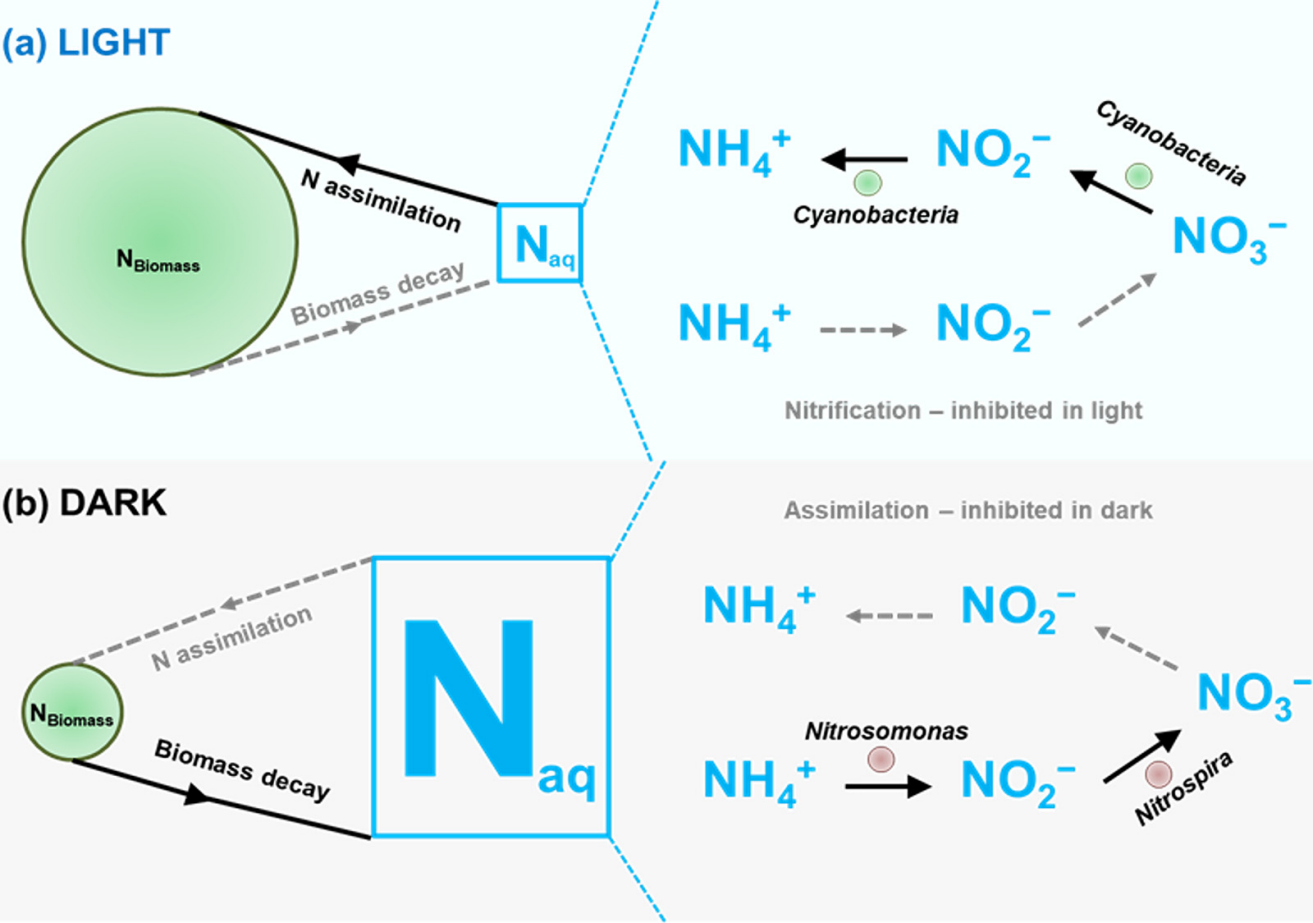
Model depicting the process of nitrate accumulation in the surface water stored in subsurface condition. (a) In the presence of light, the decay of biomass is low, resulting in lower concentration of free nitrogen in the water. In addition, the nitrate that is present or introduced into the system is assimilated back to the biomass by nitrate assimilating *Cyanobacteria*. (b) In the dark, the decay of biomass is high, resulting in high concentration of free N in the water. *Nitrosomonas* and *Nitrospira* are dominant in the dark and are involved in converting the free N to nitrate. The nitrate in the water is not assimilated back into the biomass due to the low abundance of nitrate assimilating *Cyanobacteria*.

Based on our results, we propose a mechanistic model to explain the accumulation of nitrate in the surface water stored in dark conditions (Fig. 5). This model brings together different dynamics of known microbial nitrogen transformation processes (28) that occur under light and dark conditions to provide an explanation for the water quality changes observed when surface water is stored in the dark.

In the light, the decay rate of the biomass was low, and the total algae content remained relatively high throughout the experiment. This may have resulted in relatively lower concentration of free nitrogen in the water. In addition, *Synechococcus* and other nitrate assimilating bacteria were dominant in the light. Hence, free nitrogen introduced into the water by the decay of biomass was likely assimilated back into the biomass (29). In the dark, total algae depleted rapidly due to the inhibition of photosynthesis. This decay of biomass led to the introduction of free nitrogen in the water. High abundance of nitrifying bacteria (*Nitrosomonas* and *Nitrospira*) facilitated the conversion of free or soluble nitrogen compounds to nitrate. The low abundance of nitrate assimilating bacteria in the dark may have ensured that the nitrate was not assimilated back into the biomass.

Taken together, our study shows that the storage of raw surface water in the dark results in an accumulation of nitrate in the water. The accumulation is likely due to an increase in nitrification rate and a decrease in the assimilation rate of nitrate back into the biomass. Our findings suggest that nitrate should be closely monitored if raw surface water is to be stored in the dark and that downstream treatment to remove nitrate may be required.

## MATERIALS AND METHODS

### Sampling and experimental conditions.

Water samples were collected from a freshwater reservoir in Singapore on 9th October 2015, 11:30 a.m. local time. The reservoir is part of a water catchment system that collects rainwater before it is treated and distributed as drinking water. Water quality changes of reservoir water stored in the dark would thus be an accurate indication of water quality changes that may occur when surface water’s access to light is restricted before treatment.

Water samples were collected from the surface (<1 m depth) of the reservoir using a van Dorn water sampler (Wildco Instruments, Wildlife Supply Company, USA). The samples were immediately transferred to 20-L transparent Nalgene polycarbonate carboys with spigots (ThermoFisher Scientific, Waltham, MA) and transported to the laboratory. The filling of the carboys was randomized.

Three LED light sources (6500 K, 20 W, 1600 lumens) were used to illuminate each of the three carboys containing the sample, while three other carboys were covered with opaque black polyethylene bags to simulate the dark subsurface condition. Spectrum for the light source was measured using a Lisun Portable color spectroradiometer and falls mainly in the visible spectrum (380–750 nm; Fig. S1). The light sources were placed approximately 30 cm from the center of the carboys. To monitor changes in water quality, 100 mL of water was collected at monthly intervals from each carboy through the spigot. To investigate the influence of the microorganisms in water quality changes when the reservoir water is kept in the dark, two carboys containing reservoir water were sterilized by autoclaving at 121°C for 20 min. One carboy served as the abiotic control for the water exposed to light while the other served as the abiotic control for the water stored in the dark.

The carboys are made of clear polycarbonate and they do not significantly block light in the visible region of the spectrum, which is the source of energy for photosynthesis, which all organisms in the ecosystem depend on. As visible light is a major factor that shapes the microbial communities that may influence water quality, the study aims to test the effects of the absence of light (darkness) on water quality. Thus, *c*ontinuous illumination of control samples was chosen to achieve maximum differences between light and dark samples over the duration of the experiment. Continuous light treatment does not adversely impact cyanobacterial population and diversity (30).

### Measurement of water quality parameters.

Turbidity, pH, ORP, and chloride were measured *in situ* using an EXO2 multiparameter sonde fitted with turbidity and pH/ORP sensor as well as a chloride ion-selective electrode (Xylem Analytics, Hemmant, Australia). Total soluble organic carbon (TOC_aq_), total soluble inorganic carbon (TIC_aq_), total soluble carbon (TC_aq_) and total soluble nitrogen (TN_aq_) in the water samples were quantified using a TOC-L Analyzer (Shimadzu Corporation, Kyoto, Japan) after the samples were filtered through hydrophilic PVDF syringe filters (pore size 0.2 μm, ThermoFisher Scientific, Waltham, MA). The original water samples before filtration were used to measure the composite TOC, TIC, TC, and TN values using the same TOC-L analyzer. The concentrations of nitrate, nitrite, and ammonia in the water were determined using the Hach Kits (Hach Company, Loveland, CO) “Nitrate TNTplus Vial Test, LR (0.2-13.5 mg/L NO_3_-N),” “Nitrite TNTplus Vial Test, LR (0.015-0.600 mg/L NO_2_-N)” and “Ammonia TNTplus Vial Test, ULR (0.015-2.00 mg/L NH_3_-N),” respectively. TIN_aq_ was determined by adding the concentrations of nitrate, nitrite, and ammonia in the water. TON_aq_ was estimated by subtracting TIN_aq_ from TN, which was obtained along with the TC, IC, and TOC from the TOC-L series analyzer. HPCs for the water samples were conducted using the drop-plate CFU method as described elsewhere (31). Total algal content in terms of the chlorophyll *a* concentration in the water was measured *in situ* using the EXO2 multiparameter sonde fitted with EXO Total Algae Sensor. The rates of change were expressed as “mean ± standard deviation.” One-way analysis of variance (ANOVA) at a 95% confidence interval (*P* < 0.05) was used to determine the statistical significance of the observed differences among the mean values.

### RNA extraction and sequencing.

Total RNA was extracted from the water samples using the PowerWater RNA isolation kit (Mo Bio Laboratories Inc, Carlsbad, USA) according to manufacturer’s instructions. Briefly, at the end of the 6-month experimental period, 5 L of the water samples were filtered through nitrocellulose filters (diameter: 47 mm, pore size: 0.2 μm). Then the filters were transferred into the PowerWater Bead Tubes along with warm lysing solution at 55°C. The tubes containing the filters were vortexed vigorously for 5 min. Thereafter, the tubes were centrifuged and the supernatants were carefully transferred to silica membranes on spin filters for RNA binding. The spin filters were centrifuged to discard the flow through containing non-RNA components and then DNase I was added to the spin filters to remove the genomic DNA. RNA was eluted in highly pure RNase free water and quantified by Invitrogen Qubit fluorometric quantitation (Thermo Fischer Scientific, Waltham, MA). The RNA samples were sequenced using the PCR-free paired end sequencing approach on an Illumina MiSeq sequencing by synthesis (SBS) platform.

### RNA sequencing data analyses.

The sequences were uploaded to MG-RAST server version 3.0 (32) under project ID “LD Transcriptomes”. Reads were pre-processed by “SolexaQA” (33) to trim low-quality regions, and artificial replication reads were analyzed and removed using “duplicate read inferred sequencing error estimation” (DRISEE) (34). The sequences were screened for contamination using “Bowtie2” (35) against H. sapiens NCBI v36 as reference database. An initial search using “VSEARCH” (36) against a reduced RNA database, which is a 90% identity clustered version of SILVA, Greengenes, and RDP databases, was used for rRNA detection. The reads were clustered at 97% identity using CD-HIT (37) and the longest sequence was picked as the cluster representative. BLAT (38) similarity search for the longest cluster representative sequences was performed against an RDP database with a cut-off E-value of 1E-10, minimum identity of 97% and a minimum alignment of 50 bp. The OTU table was generated and filtered to exclude eukaryotic and chloroplast sequences as well as sequences from unidentified domains and only prokaryotic sequences were retained. Subsequent analyses were performed in R as described elsewhere (39). Data were analyzed using “vegan” (40) and “phyloseq” (41) packages. For the comparison of OTU abundances across the samples, normalized OTU tables were used. All samples were normalized against the total OTU abundance of individual samples. To compare the OTU abundances among the groups of samples, the Welch’s test statistics were used. Functional annotation of taxa was performed using the program “functional annotation of prokaryotic taxa” (FAPROTAX) on the normalized OTU table (42). The predicted abundances of functions among the groups of samples were also compared using Welch’s test statistics. Significant differences in the mean values were calculated at the 95% confidence interval (*P* < 0.05). Bonferroni correction was applied where appropriate to the control for the effect of testing multiple hypotheses simultaneously.

### Quantification of ammonia oxidizing bacteria and *Nitrospira* using RT-qPCR.

Synthesis of cDNA from the RNA samples was performed using the Invitrogen SuperscriptIII First-Strand Synthesis Supermix (ThermoFisher Scientific, Waltham, MA). qPCR was performed using the KAPA SYBR Fast Universal 2× qPCR Master Mix (Kapabiosystems) on a StepOne Plus real-time PCR system. PCR thermal cycling was carried out by using an initial denaturation step of 95°C for 20 s, followed by 40 cycles of denaturation at 95°C for 1 s and annealing/extension at 60°C for 20 s (43). Forward primers (GGAGRAAAGCAGGGGATCG and GGAGGAAAGTAGGGGATCG) at a weight ratio of 2:1 and a reverse primer (CTAGCYTTGTAGTTTCAAACGC) were used to amplify the 16S rRNA gene of ammonia oxidizing bacteria (44). The 16S rRNA gene of *Nitrospira* sp. was amplified using the forward primer CCTGCTTTCAGTTGCTACCG and reverse primer GTTTGCAGCGCTTTGTACCG (44).

### Culture and maintenance of Nitrosomonas europaea and *Nitrobacter* sp.

A model ammonia-oxidizing bacterial strain Nitrosomonas europaea Winogradsky (ATCC 19718) was obtained from the American Type Culture Centre (ATCC) and maintained on mineral salt growth medium (10 mM NaCl, 0.4 mM KH_2_PO_4_, 1 mM KCl, 1 mM CaCl_2_·2H_2_O, 1 mM NH_4_Cl, 0.2 mM MgSO_4_, 1 mL trace element solution, 0.0002% bromothymol blue, and 200 mM HEPES) as described in a previous report (45). Experimental cultures were prepared by inoculating 1% (vol/vol) of *N. europaea* cultures into fresh mineral salt growth medium broth and incubating at 25°C. A model nitrite-oxidizing bacterial strain *Nitrobacter* sp. (ATCC 51922) was also obtained from the ATCC and maintained on mineral medium (0.1 mM CaCO_3_, 0.9 mM NaCl, 0.2 mM MgSO_4_.7H_2_0, 1.1 mM KH_2_PO_4_, 1 mM NaNO_2_, and 1 mL trace element solution) (46). Experimental cultures were prepared by inoculating 1% (vol/vol) of *Nitrobacter* sp. cultures into fresh mineral NOB medium and incubating at 25°C. Cultures of *N. europaea* and *Nitrobacter* sp. were resuspended in the respective mineral salt growth media (1% vol/vol). Before the experiment, *N*. *europaea* and *Nitrobacter* sp. cultures were grown to the late exponential phase. Cultures were diluted in a 1:10 ratio and 25 mL of each diluted bacterial culture was transferred into 50-mL transparent polystyrene tubes. An LED light source (6500 K, 100 W) was used to illuminate three tubes each of *N*. *europaea* and *Nitrobacter* while three tubes each of *N*. *europaea* and *Nitrobacter* were covered with aluminum foil and kept in the dark. Nitrate, nitrite, and ammonia were quantified on a weekly basis using the Hach water quality testing kits (Hach Company, Loveland, CO). All experiments were performed in triplicate.

### Data availability.

Raw merged sequence files are available in the Sequence Retrieval Archive under the BioProject accession PRJNA781425.
