# Supplementary material for: The Dark Side of Microbial Processes: Accumulation of Nitrate During Storage of Surface Water in the Dark and the Underlying Mechanism
Source: Microbiol Spectr. 2022 Jan 5;10(1):e02232-21. doi: 10.1128/spectrum.02232-21 (PMC8729765; doi:10.1128/spectrum.02232-21)
Supplement: SUPPLEMENTAL FILE 1 — Supplemental material. Download SPECTRUM02232-21_Supp_1_seq1.pdf, PDF file, 0.1 MB [file spectrum02232-21_supp_1_seq1.pdf]

Supplementary Information for

The Dark Side of Microbial Processes: Accumulation of Nitrate during Storage of Surface Water in the Dark and the Underlying Mechanism

Amit Kumar [1,2#], Daphne H.P. Ng [1,2#], Sakcham Bairoliya[1,2] and Bin Cao *[1,2]

[1]School of Civil and Environmental Engineering, Nanyang Technological University, 50 Nanyang Avenue, Singapore 639798

[2]Singapore Centre for Environmental Life Sciences Engineering, Nanyang Technological University, 60 Nanyang Drive, Singapore 637551

*Corresponding author: Dr. Bin Cao (@embb_ntu), School of Civil and Environmental Engineering, 50 Nanyang Ave, N1-01C-69, Nanyang Technological University, Singapore, E-mail: bincao@ntu.edu.sg, Tel: +65 6790 5277, Fax: +65 6791 0676.

[#]These authors contributed equally to this work.

Pages: 3

Tables: 1

Figures: 1

Table S1: Sequence statistics for all samples based on raw and post quality control files.

Parameter	Light_1	Light_2	Light_3	Dark_1	Dark_2	Dark_3
**Upload: bp Count**	406,962,267	371,858,269	419,684,481	389,895,599	364,864,402	394,181,609
**Upload: Sequences Count**	2,475,763	2,306,771	2,564,387	2,354,574	2,208,405	2,397,406
**Upload: Mean Sequence Length**	164 ± 28 bp	161 ± 25 bp	164 ± 27 bp	166 ± 29 bp	165 ± 29 bp	164 ± 29 bp
**Upload: Mean GC percent**	51 ± 6 %	50 ± 6 %	51 ± 6 %	54 ± 5 %	53 ± 5 %	53 ± 5 %
**Artificial Duplicate Reads: Sequence Count**	1,179,756	1,129,112	1,232,255	852,182	801,185	901,318
**Post QC: bp Count**	176,861,324	158,640,270	184,267,846	202,863,100	197,326,647	197,646,181
**Post QC: Sequences Count**	1,139,122	1,046,263	1,188,924	1,310,355	1,265,614	1,297,218
**Post QC: Mean Sequence Length**	155 ± 43 bp	152 ± 40 bp	155 ± 41 bp	155 ± 43 bp	156 ± 42 bp	152 ± 43 bp
**Post QC: Mean GC percent**	51 ± 6 %	50 ± 7 %	51 ± 6 %	53 ± 5 %	53 ± 5 %	53 ± 5 %
**Processed: Predicted rRNA Features**	606,561	569,606	629,612	694,869	686,582	702,205
**Alignment: Identified rRNA Features**	424,568	387,826	435,841	423,996	416,784	437,924

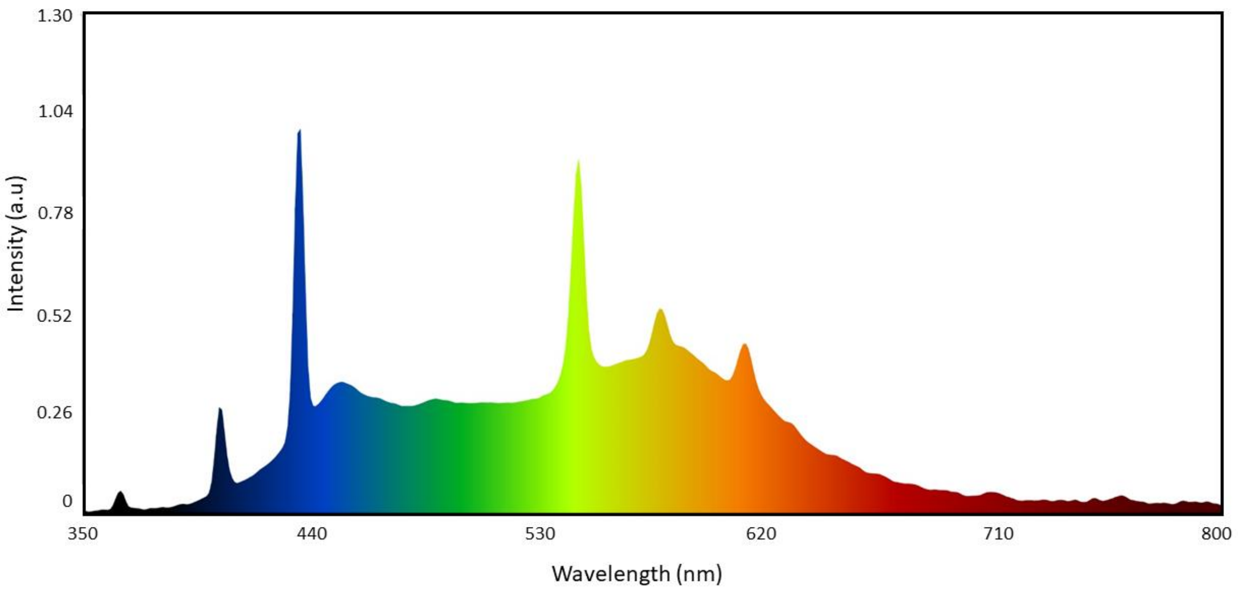

Figure S1: Spectrum of the light source used.
